# Parenting stress and pandemic burden in families with crying, sleeping, and feeding problems during COVID-19: a case-control study

**DOI:** 10.1186/s40359-025-02714-z

**Published:** 2025-04-17

**Authors:** Michaela Augustin, Bernhard Haller, Volker Mall, Ina Nehring, Maria Licata-Dandel, Anna Friedmann

**Affiliations:** 1 TUM School of Medicine and Health, Social Pediatrics, Technical University of Munich, Munich, Germany; 2 German Center for Child and Adolescent Health (DZKJ), partner site Munich, Munich, Germany; 3 TUM School of Medicine and Health, Institute of AI and Informatics in Medicine, Technical University of Munich, Munich, Germany; 4 kbo-Kinderzentrum Munich, Munich, Germany; 5 Department of Psychology, Charlotte Fresenius University, Munich, Germany

**Keywords:** Child, Infant, Crying, Sleeping, Feeding, Regulatory problems, Pandemic, COVID-19, Parenting stress, Pandemic burden

## Abstract

**Background:**

Families with child crying, sleeping, and feeding problems are a vulnerable group due to high levels of parenting stress and an increased risk for child abuse. However, little is known about their specific situation during the COVID-19 pandemic. The aim of our study was to assess parenting stress and pandemic burden / constraints in families with child crying, sleeping, and feeding problems (= clinical sample) compared to a non-clinical sample. First, we hypothesized that parenting stress during the pandemic would be higher in the clinical sample compared to the nonclinical sample. Moreover, we aimed to explore the extent to which pandemic-related burden / constraints were perceived as restrictive and whether they differed between the two groups. Last, we intended to explore which pandemic-related constraints were associated with parenting stress during the pandemic, controlled for demographic variables.

**Methods:**

Using a case-control matched design, standardized questionnaire data from *N* = 142 parents of children aged 0–24 months, drawn from two different studies (clinical sample: *n* = 71, nonclinical sample: *n* = 71) were analyzed. Groups were compared using paired sample t-tests and variables associated with parenting stress were analyzed using a multiple linear regression model.

**Results:**

Parenting stress (*p* <.001, *d*_*z*_=0.64) and overall pandemic burden (*p* =.034, *d*_*z*_=0.26) were higher in the clinical group compared to the nonclinical group, whereas groups did not differ significantly in terms of specific pandemic-related constraints. Both groups reported being burdened especially by restricted leisure activities (79.6%) and reduced family support services (74%). Parenting stress was associated with child age (β=-0.20, *p* =.024), the presence of siblings (β=-0.21, *p* =.034), overall pandemic burden (β = 0.25, *p* =.009), increased childcare responsibilities (β = 0.19, *p* =.046), and increased family conflicts (β = 0.29, *p* <.001).

**Conclusions:**

Families with crying, sleeping, and feeding problems experienced high levels of parenting stress and were significantly burdened by the pandemic itself. Thus, as a double-risk group during the pandemic, they require targeted attention in prevention and intervention efforts, including access to professional support and opportunities for safe social connection and leisure activities.

**Trial registration:**

Clinical group: German Register of Clinical Studies DRKS00019001, registration date: 2020-01-28, nonclinical group: OSF, https://osf.io/tksh5, registration date: 2021-01-15.

**Supplementary Information:**

The online version contains supplementary material available at 10.1186/s40359-025-02714-z.

## Introduction

The COVID-19 restriction measures, accompanied by social isolation, limited access to support services, challenges due to additional childcare responsibilities and economic changes, affected parent and child well-being [1, 2, 3, 4]. An increase in parental mental health problems, especially depression and anxiety symptoms, as well as more parent-child relationship problems were reported [5, 6, 7]. Mental health deterioration among children from preschool age has also been well established [8], including among German children [9, 10]. However, although highly prevalent, psychological problems in infants and toddlers–i.e., crying, sleeping, and feeding problems- have been less focused in pandemic-related research. Even before the pandemic, these problems occurred very frequently, with approximately 20 to 30% of infants affected, and up to 10% diagnosed with a clinical disorder [11, 12, 13, 14, 15, 16]. Results from the German CoronabaBY study found increased rates of infant crying/sleeping problems of up to 35.5% during the pandemic [17]. Perez et al. [18] reported increased infant crying and sleeping, but not feeding problems in a German pandemic sample compared to a pre-pandemic sample. Additionally, evidence from the Netherlands showed that outpatient clinic visits and admission rates of children with excessive crying increased during the pandemic [19].

Due to the challenging symptomatology, parents of children with crying, sleeping, and feeding problems are generally at risk of experiencing very high levels of parenting stress [20, 21, 22, 23]. Parenting stress is defined as an adverse psychological reaction to an imbalance between childcare demands and parental resources [20, 24]. Since it is associated with lower parental emotional availability, more harsh and disciplining parenting behavior and a higher risk of child maltreatment and neglect [25, 26, 27], parenting stress affects the whole family system. This is problematic in nonclinical populations, but of great concern in clinically burdened families, e.g., in families experiencing child crying, sleeping, and feeding problems: Even before the pandemic, these families were known to bear a risk of very stressful and overexerting experiences which can lead to potentially life-threatening incidents of child maltreatment, e.g., by shaking the child [28, 29, 30].

During the course of the pandemic, parenting stress generally increased significantly in families with young children [7, 17, 31]. A meta-analytic review by Chung et al. [32] confirmed that, during the pandemic, parenting stress was related to more child and parental mental health problems, parent-child relationship problems, and reduced parental engagement. Additional stress factors, as evident during the pandemic [7], could be detrimental to parental functioning, with possible severe implications for parenting behavior. Large German studies indicate that families with infants and toddlers were burdened by the pandemic [7, 33]. In the CoronabaBY study, perceived overall pandemic burden as well as increased conflicts in the family were moderately associated with parenting stress [7]. Clemens et al. [34] found that a decrease in parental satisfaction with the sharing of childcare duties during COVID-19 was associated with stronger concerns about potentially harmful parenting behavior such as physical violence, yelling at the child, impatience, and poorer coping of the pandemic.

Studies indicate that parents of children with special care needs, such as neurodevelopmental disorders have been particularly challenged by the pandemic compared to the pre-COVID period [35]. While special care needs also apply to children with early crying, sleeping, and feeding problems, specific research on the role of parenting stress in these problems during the pandemic is scarce. Buechel et al. [7] found moderate positive correlations between parenting stress and child crying/sleeping as well as feeding in children aged 0–3 years in a general sample during the pandemic. Another study found associations between perceived general self-stress and infant sleeping and crying problems in the first year of life during the pandemic [36]; however, the assessment of stress in this study did not specifically focus on parenting stress.

Although it is conceivable that, during the pandemic, parenting stress was higher in parents of children with crying, sleeping, and feeding problems compared to nonclinical families, there is a lack of data investigating this assumption. Furthermore, little is known about their additional burden pertaining the pandemic-related constraints compared to families without these problems. To our knowledge, this is the first study comparing families of a clinical sample (crying, sleeping, and feeding problems) with a nonclinical group regarding parenting stress and pandemic-related burden during the pandemic. Additionally, no study has examined the role of specific pandemic-related constraints on parenting stress, controlling for potentially demographic confounders and taking into account a clinical sample. Since parenting stress is a risk factor for maltreatment [37, 38, 39] and is associated with both child and parent mental health problems [32, 40], it is necessary to gain a deeper understanding of this construct in order to derive appropriate prevention and intervention strategies. Consequently, investigating potential stressors for the highly prevalent and vulnerable group of families with child crying, sleeping, and feeding problems is crucial for preventing several short- and long-term consequences.

Thus, the aim of our study was to assess parenting stress and pandemic burden in families with infant crying, sleeping, and/or feeding problems compared to a nonclinical sample. First (I), we assumed to find higher parenting stress (primary outcome) during the pandemic in parents of children with crying, sleeping, and/or feeding problems (= clinical sample) compared to a nonclinical sample. Additionally (II), we aimed to explore the extent to which overall pandemic burden and specific pandemic-related constraints were perceived as restrictive by the clinical compared to the nonclinical sample (IIa), and whether the overall pandemic burden and specific pandemic-related constraints differed between the clinical compared to the nonclinical sample (IIb). Last (III), we intended to explore which pandemic-related constraints were associated with parenting stress during the pandemic, controlled for demographic variables.

## Methods

### Study design

A retrospective case-control matched design comparing data from two different studies was conducted. Data were collected between December 2020 and March 2022. Both studies were approved by the Ethics Committee of the Technical University of Munich (app-based intervention study: vote number 56/18S, CoronabaBY study: vote number 322/20S) and were pre-registered (app-based intervention study: German Register of Clinical Studies DRKS00019001, registration date: 2020-01-28; CoronabaBY study: OSF, https://osf.io/tksh5, registration date: 2021-01-15). The content of this manuscript is presented in accordance with the STROBE guidelines for observational and case-control studies [41].

### Participants

The clinical group was recruited within the framework of a clinical RCT [42]. In a cry baby outpatient clinic, participants were screened for eligibility. The target group was German-speaking parents of children aged 0–24 months who had contacted a cry baby outpatient clinic in Southern Germany for a first consultation due to child crying, sleeping and/or feeding problems. Interested parents were referred to the study team. After verbal study information and consent, participants postally received the study information, the declaration of consent as well as paper-pencil questionnaires, which they returned postally to the study team after completing them. Between December 2020 and January 2022, pandemic burden questionnaires were sent to the participating parents in addition to their regular questionnaires for the RCT study, which were included in this paper (*n* = 71). For the nonclinical comparison group, a case-control matched subsample (*n* = 71) from the CoronabaBY study [7], which was recruited from February 2021 to March 2022, was included. To avoid confounding of demographic variables for group comparisons, cases were matched 1:1 in terms of parental age (tolerance 5 years), participating parent (father/mother), nationality, education (qualified for university admission/other), single-parent status, child age (tolerance 6 months), child sex and siblings (yes/no), because these variables were observed to be related to study outcomes [31, 43, 44]. In the CoronabaBY study, participants were recruited via the smartphone app ‘Mein Kinder- und Jugendarzt’ (‘My pediatrician’) (www.monks-aerzte-im-netz.de), which is used as a communication tool for parents and their pediatrician. First, pediatricians in Bavaria (Southern Germany) were invited to participate in the study. After giving consent, all patients of the participating pediatricians received an invitation for study participation via app. All study information and the informed consent form were provided via the app. 

### Measures

#### Pandemic-related burden / constraints

Perceived overall pandemic burden was assessed with the item ‘Taken together, what do you think: how stressful is/was the COVID-19 pandemic for you (please think of measures like social restrictions but also your personal experiences, related worries etc.)?’. Answers were based on a 5-point Likert scale (from 1 = not at all stressful to 5 = very stressful). Furthermore, eight items were asked about specific pandemic-related constraints (e.g.,‘How restricted are your private social contacts currently (e.g., with family, friends, acquaintances)?’): restricted social contacts of the parents, restricted social contacts of the child, reduced family support services, restricted leisure activities, increased childcare responsibilities, increased family conflicts, worries about COVID-19 infections, and financial burden due to COVID-19. Financial burden due to COVID-19 was assessed using a 4-point Likert scale, all other items were assessed using 5-point Likert scales from 1 = not at all/ /not restricted at all to 5 = very much / very restricted. An overview of the items is provided in the supplement (Table S1). The questionnaire has been used in previous studies [7, 17, 45], where weak to moderate significant correlations between the perceived overall pandemic burden and the specific pandemic-related constraints as well as between the pandemic-related items with parenting stress, child and parental psychopathology were identified [7]. For this study, correlations between the pandemic-related items, separated by group, can be found in the supplement (Table S2).

#### Parenting stress

Parenting stress was assessed with the Eltern-Belastungs-Inventar (EBI) [46], which is a German adaptation of the Parenting Stress Index [47]. The questionnaire is based on 48 items covering the parent domain (impairment of parental functions) and the child domain (stress emanating from the child’s behavior). Subscale ratings, overall scores separately for the child and parent domain, and a total score can be built. In this study, the overall parent domain score (based on the subscales attachment, isolation, parental competence, depression, health, role restriction, and spouse-related stress) was used as an outcome variable. Answers were based on a 5-point Likert scales (1 = strongly agree to 5 = strongly disagree). Since the normative values differed between parents in partnerships and those not in partnerships, we used T-scores to ensure comparability. Clinical cut-offs for the EBI parental domain are T > 60 for highly stressed and T > 70 for very highly stressed. The parent domain has shown to have an excellent internal consistency (Cronbach α = 0.93). External validity has been proven in validation studies using different samples [48].

#### Sociodemographic data

In both studies, parental age, participant’s relation to child, educational qualifications, nationality, first language, partnership status, as well as child’s age and sex, and siblings were assessed. Furthermore, information about the child’s symptoms was assessed in the clinical group.

#### Statistical analyses

To examine group differences in the parenting stress score (confirmatory hypothesis I) as well as in overall pandemic burden and specific pandemic-related constraints between the clinical and the nonclinical sample (exploratory question IIb), paired sample t-tests were used considering matched individuals as paired observations. In this model, given our sample size, large, medium and small effects could be detected with sufficiently high probability (1-β = 0.99 for large and medium effects and 1-β = 0.38 to 0.99 for small effects). For reporting descriptive statistics of how burdening parents perceived the pandemic and specific constraints (question IIa), percentages of 4 and 5 point-Likert scale answers (3 and 4 point-Likert scale answers for financial burden) were summarized as ‘(very) burdened’ or ‘(very) restricted’. To detect if parenting stress was associated with pandemic-related constraints, a multiple linear regression model was fitted to the data. Parenting stress was used as the outcome variable and overall pandemic burden as well as specific pandemic-related constraints were included as predictors. Sample (clinical = 0, nonclinical = 1) was included as a predictor to test for robustness of the results of the previous t-test for differences between both groups regarding parenting stress, controlling for the other variables included in the regression model. The demographic variables parental age, educational qualifications (qualified for university admission = 1, other = 0), nationality (German = 0, other = 1), partnership status (single parent = 0, not single parent = 1), child’s age and sex (girl = 0, boy = 1), and siblings (no = 0, yes = 1) were entered as covariates. Analyses were based on α = 5% (two-tailed) and were performed in IBM SPSS Statistics Version 28.0 [49]. Power for detectable effects was calculated using the GPower 3.1 software [50].

## Results

### Sample characteristics

A total of 142 parents (*n* = 71 in both groups) were included. Participants (mean age 34.09, SD 4.20 years) were predominantly mothers (138, 97.2%) of German nationality (122, 85.9%) with higher education (120, 84.5% qualified for university admission). Children (78, 53.5% boys) were on average 9.49 (SD 5.76) months old and predominantly had no siblings (98, 69.0%). In the clinical sample, most parents reported that they had consulted the outpatient clinic due to sleeping problems (30, 42.3%), followed by combined crying, sleeping and/or feeding problems (24, 33.8%). The demographics divided by group are presented in Table 1.

**Table 1:** Participant characteristics

Characteristics	Clinical Group (*n* = 71)	Nonclinical Group (*n* = 71)	Absolute Standardized Difference
Parental age (years), mean (*SD*)	34.45 (4.01)	33.73 (4.38)	0.17
Participating parent, n (%)			
Mother	69 (97.2)	69 (97.2)	-
Father	2 (2.8)	2 (2.8)	-
Educational qualification, n (%)			
Qualified for university admission	60 (84.5)	60 (84.5)	-
Other	11 (15.5)	11 (15.5)	-
Nationality, n (%)			
German	61 (85.9)	61 (85.9)	-
Other	10 (14.1)	10 (14.1)	-
First language, n (%)			
German	61 (85.9)	66 (93.0)	0.23
Other	10 (14.1)	5 (7.0)	-
Single parent, n (%)	2 (2.8)	2 (2.8)	-
Child age (months), mean (*SD*)	9.89 (4.87)	9.08 (6.54)	0.14
Child sex, n (%)			
female	33 (46.5)	33 (46.5)	-
Male	38 (53.5)	38 (53.5)	-
Siblings, n (%)			
Yes	22 (31.0)	22 (31.0)	-
No	49 (69.0)	49 (69.0)	-
Child’s symptom duration (months), mean (*SD*) (clinical sample only)	7.28 (5.20)	-	-
Reason for consultation (clinical sample only)			
Sleeping problems	30 (42.3)	-	-
Feeding problems	9 (12.7)	-	-
Crying, whining	5 (7.0)	-	-
Combined problems	24 (33.8)	-	-
Other	3 (4.2)	-	-

### Parenting stress

In the clinical group, the EBI parenting stress mean T-score was 63.66 (*SD* = 8.04) and 71.4% of the parents reported a score above the clinical cutoff-value of T = 60. In the nonclinical group, the EBI parenting stress mean T-score was 56.39 (*SD* = 9.29) and 33.8% of the parents reported a score above the clinical cutoff-value. Parents in the clinical sample scored significantly higher on the EBI than parents in the nonclinical sample, *t*(69) = 5.37, *p* <.001, *d*_*z*_=0.64, *95%*,* CI* [0.38 to 0.90].

### Overall pandemic burden and specific pandemic-related constraints

**Fig. 1:**
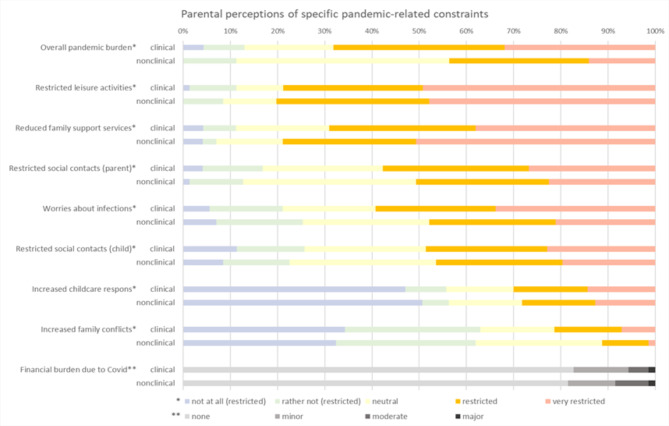
Parental perceptions of specific pandemic-related constraints

In total, 54.9% of the parents reported to be (very) burdened by the pandemic. Regarding specific pandemic-related constraints, most parents felt (very) restricted with regard to leisure activities (79.6%), followed by family support services (74%), social contacts of the parents (54.2%), worries about infections (53.6%), social contacts of the child (47.5%), increased childcare responsibilities (29.1%), increased family conflicts (16.4%) and financial burden (6.2%). Percentages divided per groups are displayed in Fig. 1.

The clinical group reported a significantly higher overall pandemic burden (*M* = 3.83, *SD* = 1.11) compared to the nonclinical (*M* = 3.49, *SD* = 0.87) group, *t*(68) = 2.16, *p* =.034, *d*_*z*_=0.26, *95%*,* CI* [0.02 to 0.50]. No differences were found in terms of specific pandemic-related constraints (Table 2).

**Table 2:** Analysis of group differences between clinical and nonclinical sample regarding pandemic Burden / Constraints (Paired sample t-Tests)

Variable	M_clin_ (SD)^a^	M_nonclin_(SD)^a^	t	*p*	d_z_	95% CI	*N*_matched pairs_
Overall pandemic burden	3.83 (1.11)	3.49 (0.87)	2.16	.034^b^	0.26	[0.02, 0.50]	69
Restricted leisure activities	4.15 (1.05)	4.20 (0.95)	-0.24	0.809	− 0.03	[0.02, 0.50]	71
Reduced family support services	3.92 (1.12)	4.18 (1.06)	-1.38	0.171	− 0.16	[-0.40, 0.07]	71
Restricted social contacts (parent)	3.63 (1.14)	3.59 (1.01)	0.21	0.837	0.02	[-0.21, 0.26]	71
Worries about infection	3.66 (1.25)	3.37 (1.21)	1.44	0.153	0.17	[-0.06, 0.41]	71
Restricted social contacts (child)	3.34 (1.30)	3.36 (1.20)	-0.07	0.949	− 0.01	[-0.24, 0.23]	70
Increased childcare responsibilities	2.41 (1.55)	2.36 (1.53)	0.24	0.811	0.03	[-0.21, 0.26]	70
Increased family conflicts	2.31 (1.28)	2.18 (1.05)	0.77	0.446	0.09	[-0.14, 0.32]	70
Financial burden	1.21 (0.51)	1.29 (0.67)	-0.85	. 400	− 0.10	[-0.34, 0.17]	68

^a^based on 5-point Likert scales from 1 = not at all/not at all restricted to 5 = very much/very restricted (4-point Likert scale for financial burden).

^b^statistically significant based on α = 0.05, 2-tailed

**Table 3:** Prediction of parenting stress (multiple regression)

Predictor	B	β	SE	*p*	95% CI
Sample (clinical/nonclinical)	-6.32	− 0.33	1.48	<.001^a^	[-9.26, -3.39]
Parental age	0.26	0.12	0.22	0.225	[-0.16, 0.69]
Educational qualification (qualified for University admission/other)	-0.80	− 0.03	2.18	0.715	[-5.10, 3.51]
Nationality (German/other)	-1.26	− 0.05	2.38	0.598	[-5.97, 3.45]
Single parent (yes/no)	2.80	0.05	4.37	0.522	[-5.85, 11.46]
Child age	-0.32	− 0.20	0.14	.024^a^	[-0.60, -0.04]
Child sex (female/male)	1.13	0.06	1.49	0.448	[-1.81, 4.08]
Siblings (yes/no)	-4.37	− 0.21	2.04	.034^a^	[-8.41, -0.32]
Overall pandemic burden	2.37	0.25	0.89	.009^a^	[0.60, 4.13]
Restricted leisure activities	1.39	0.15	0.95	0.147	[-0.50, 3.28]
Reduced family support services	0.18	0.02	0.88	0.840	[-1.57, 1.92]
Restricted social contacts (parent)	-0.73	− 0.08	1.06	0.496	[-2.83, 1.38]
Worries about infection	-0.83	− 0.11	0.65	0.206	[-2.11, 0.46]
Restricted social contacts (child)	-1.53	− 0.20	0.84	0.071	[-3.19, 0.13]
Increased childcare responsibilities	1.21	0.19	0.60	.046^a^	[0.02, 2.39]
Increased family conflicts	2.43	0.29	0.72	< 0.001 ^a^	[1.01, 3.85]
Financial burden	-1.87	− 0.13	1.20	0.121	[-4.25, 0.50]

^a^statistically significant based on α = 0.05, 2-tailed

### Associated factors of parenting stress

A multiple regression model (*N* = 133) showed that parenting stress was negatively associated with child age (*p* =.024) and the presence of siblings (*p* =.034), and positively associated with overall pandemic burden (*p* =.009), burden due to increased childcare responsibilities (*p* =.046), and burden due to increased family conflicts (*p* <.001), F(17,115) = 4.22, *p* <.001, *R*^*2*^_*adj*_ = 0.29 (Table 3).

## Discussion

### Principal findings

To the best of our knowledge, this is the first study examining parenting stress and pandemic-related burden / constraints in families with child crying, sleeping, and feeding problems. The majority of the affected parents scored above the clinical cutoff score for highly stressed and they reported significantly higher levels of parenting stress during the pandemic compared to a nonclinical group. In total, the majority of the parents reported to be burdened by the pandemic, with parents of children with crying, sleeping, and feeding problems describing a significantly higher pandemic burden than the nonclinical group. Regarding specific pandemic-related constraints, parents in both groups reported to be burdened especially by restricted leisure activities, reduced family support services, restricted social contacts and worries about infections. Parenting stress was positively associated with a younger child age, the absence of siblings, a greater perceived overall pandemic burden, burden due to increased childcare responsibilities and burden due to increased family conflicts.

As expected, parenting stress was higher in parents of children with crying, sleeping, and feeding problems during the pandemic compared to a nonclinical sample, with robust results controlling for demographic variables and pandemic-related constraints. In our study, due to the lack of a longitudinal design, we could not compare the parenting stress levels to pre-pandemic scores. However, various pre-pandemic studies showed high parenting stress for parents of children with crying, sleeping, and feeding problems [21, 22, 23], and research indicates that parenting stress generally increased in the course of the pandemic [17]. Consequently, although we could assume that parenting stress might have increased in both groups during the pandemic, we still found a noticeable difference in parenting stress between families with crying, sleeping, and feeding problems compared to the nonclinical sample. In our clinical group, the parenting stress scores were in a clinically high range in about 71% of the parents and significantly higher than in the nonclinical group with a medium effect size. Medium-sized associations between parenting stress and child symptoms were also found in pre-pandemic studies [23, 51]. Thus, it is reasonable that the increased stress due to the child’s symptoms continued to be a major concern during the pandemic. This emphasizes the enduring high vulnerability of this group during the pandemic as, increased parenting stress is associated with lower parent-child interaction and an increased risk of child maltreatment [25, 26, 27].

With regard to the overall pandemic burden, the majority of parents in our study (54.9%) reported to be (strongly) burdened by the pandemic. In the nonclinical sample, the rates of 43.7% are comparable to other German pandemic-related studies, reporting rates of 50% [52] to 59% [53] for parents of children younger than 14 years. In our clinical sample, the reported pandemic burden was significantly higher. The small effect size regarding this group differences might reflect that parents of young children in general were highly affected by the pandemic, which is in line with the previously reported high rates of parents perceiving the pandemic as burdening in our and other studies [33, 52, 53]. However, although the effect size for group differences is considered small, it is important to note that a substantial proportion of 68.1% of the parents in the clinical group reported being (strongly) burdened by the pandemic. One explanation might be that these parents might have already had higher stress levels due to their child’s symptoms, which might have resulted in a reduced capacity to cope with additional perceived stress deriving from the pandemic [54]. An additional explanation could be the lack of social support during the pandemic which was found to increase parenting stress [55]. This might be especially relevant for families with crying, sleeping, feeding problems, as these parents are particularly dependent on support [56, 57]. Options to relieve the parents in their daily routine were eliminated because support systems such as grandparents or other family members were not met due to infection fears or restrictions. Third, studies showed that child crying, sleeping, and feeding problems are associated with parent psychopathology such as anxiety or depression [12, 21], and affective symptoms have increased in parents [5, 58] – especially those of very young children [59] during COVID-19. These symptoms might lead to perceptions of the pandemic as more burdening. However, we did not survey parental mental health problems in this study. As we did not test moderation or mediation effects of parental psychopathology, it would be interesting to conduct further research regarding this assumption.

Regarding specific pandemic-related constraints, restricted leisure activities, reduced family support services, restricted social contacts as well as worries about infections turned out to be the most burdening factors as reported by more than half of the parents in both groups. About 79% of the parents of children with crying, sleeping, and feeding problems reported to be (very) burdened by restricted leisure activities. To date, there are no comparable studies regarding the rates of perceived burden due to restricted leisure activities during the pandemic. However, these rates are higher compared to a large German representative national survey (Kid 0–3 study), in which slightly more than 60% of parents of young children reported boredom during the pandemic [33]. Our finding is understandable when taking into account various studies which demonstrated the importance of leisure activities for family functioning and closeness, well-being, marital and overall family satisfaction as well as child development [60, 61, 62, 63, 64, 65]. Our finding that 69% of the parents of children with crying, sleeping, and feeding problems reported to be burdened by reduced family support services emphasizes the need of providing adequate support for these families during the pandemic. The rates reported in our study are higher compared to results of the Kid 0–3 study, where about 58% of the parents of infants and toddlers were restricted by a lack of support [33]. A report by Langmeyer et al. [66] indicated the importance of contacts to professional supportive institution: In their report, 63% of the parents who had frequent contact with care institutions coped well with the pandemic. Restricted parental social contacts were reported to be burdening by 57.8% (parental contacts) and 48.6% (child contacts) of the clinical parents in our study. These rates are comparable to the Kid 0–3 study, where about 58% of parents of infants and toddlers reported to suffer from loneliness during the pandemic [33], and slightly lower compared to a large German study by Ravens-Sieberer et al. [10], focusing on older children, in which rates of 76–83% in families with children aged 11–17 years were reported. In our study, 59.2% of the parents with children with crying, sleeping, or feeding problems reported to be burdened by worries about COVID-19 infections. These rates are quite similar to rates reported in the Kid 0–3 study [33] as a well as a study by Adams et al. [67] investigating families with children aged 5 to 18 years.

With regard to factors associated with parenting stress, a multiple regression model showed that a younger child age, the absence of siblings, a greater perceived overall pandemic burden, burden due to increased childcare responsibilities and burden due to increased family conflicts were associated with higher parenting stress. The role of child age for parenting stress has so far been shown inconsistently in research. While some studies found a younger child age to be related to increased parenting stress [68], other studies especially evaluating children in the first years of life showed positive associations between age and parenting stress [7]. Kim et al. [69] described a decline of parenting stress within the first year of life and an increase in the second year. In the context of child crying, sleeping, and feeding problems, it is conceivable that the parenting stress might decrease as the child symptoms can frequently decrease after the first months of life [13]. Also, findings regarding the association between single child vs. siblings for parenting stress are heterogenous. While some studies indicated increased parenting stress with an increased number of children [70], other studies found no associations after entering additional confounders [17]. In our study, having only one child was associated with increased parenting stress. Especially in children with crying, sleeping, and feeding problems, parents often experience insecurities about to what extent child behavior (e.g. crying) is normal and helplessness about how to deal with their child [20, 71]. Additionally, during the pandemic, support offers as well as social restrictions made it difficult to exchange with other parents and professionals. This might be especially challenging for families where parents cannot compare their experiences with a second child. Further studies should investigate this assumption. Our finding that overall pandemic burden was associated with parenting stress is in line with previous research from our research group in a large sample of families (*N* = 2940) [17]. Also in line, burden due to increased family conflicts during the pandemic was positively associated with parenting stress. This indicates that the family microclimate, which was challenged during the pandemic, might be especially relevant for parental well-being and should be taken into account when offering support to burdened families. The association between burden due to increased childcare responsibilities and parenting stress might indicate that the additional responsibilities might negatively affect stress coping resources. This would be in line with the findings from Clemens et al. [34] who found that a decrease of parental satisfaction with the sharing of childcare duties during COVID-19 was associated with reduced stress coping of the pandemic. In our regression model, about 29% of the variance in parenting stress was explained by the included variables. Previous studies have also shown that other factors, such as parental mental health, family income, parental self-efficacy, infant medical risks, developmental delay, or infant temperament [72, 73, 74, 75], are associated with parenting stress and could therefore account for additional variance. However, due to the retrospective design of our study, we were unable to assess these variables. Further studies integrating these variables could complement our findings. Furthermore, given the limitations of the cross-sectional design, we could not examine trajectories or causal pathways. Thus, for instance, it cannot be excluded that parenting stress and the perception of pandemic-related constraints might be linked bidirectionally or reinforce each other. Longitudinal studies could provide more insight into this.

### Strengths and limitations

According to our knowledge, this is the first study investigating parenting stress in families with crying, sleeping, and feeding problems during the COVID-19 pandemic. Furthermore, the relevance of specific pandemic-related constraints for families with young children, and especially for families with child crying, sleeping, and feeding problems, have been neglected in research. Only a few studies have investigated how burdening specific restrictions or changes in living conditions were perceived by families at all, and most of them focused on older children. Additionally, to the best of our knowledge, this is the first study examining the role of pandemic-related constraints for parenting stress, controlling for potentially demographic confounders and taking into account a clinical sample. The study results contribute to a deeper understanding of the challenges faced by the vulnerable group of families with child crying, sleeping, and feeding problems, as well as factors associated with parenting stress. This, in turn, might contribute to the development of appropriate pre- and intervention offers to mitigate short- and long-term consequences.

However, our findings must be interpreted against the background of some limitations: First, the representativeness of the samples is limited due to the participation of mainly German-speaking, highly educated non-single-parent mothers. This is especially relevant since characteristics such as lower education status or migration background have shown to affect parental stress during the pandemic [10, 43]. Therefore, it cannot be ruled out that the levels of reported burden might be even higher populations with these characteristics. Furthermore, our clinical sample was derived from one specific clinical setting, and our nonclinical comparison group were users of the app “My pediatrician” only. Caution is necessary when generalizing our results to other clinical populations. However, the sample characteristics align with those of other studies that focus on early crying, sleeping, and feeding problems, which predominantly evaluate higher-educated families in stable partnerships [76, 77]. Future studies should include a more diverse sample to improve generalizability. Additionally, we cannot exclude the possibility that the nonclinical sample included children with crying, sleeping, or feeding problems, as this sample was not diagnosed. Furthermore, regarding post-hoc power, for the paired sample t-tests, only large to medium effects could be detected with sufficient probability, whereas the power was too low to detect small effects. Moreover, due to the limited sample size, possible differences between specific pandemic waves were not investigated. Additionally, since this data was not available for our retrospective analysis, we were unable to control for other confounding variables that might be associated with parenting stress, such as parental mental health problems for possible moderation or mediation effects. Furthermore, due to the cross-sectional design, causality cannot be inferred. Thus, for instance, the interpretation of associations between parenting stress and pandemic-related constraints should be approached with caution, as bidirectional links cannot be excluded based on our study design. Longitudinal studies would be necessary to investigate causality in this regard. Finally, we must point out that the items used to assess pandemic-related burden are not part of a validated multi-item scaled questionnaire. Although the use of such scales is generally preferable, the very rapid outbreak of the COVID-19 pandemic did not allow such processes to be carried out over a longer period of time as we attempted to integrate an evaluation of COVID-related variables at very short notice. However, the use of such self-developed single-item-based methods can be found in a large number of studies, e.g [33, 34, 78, 79]. In the study by Brown et al. [79], an additional qualitative analysis was conducted to identify categories in which parents felt challenged by the pandemic. Several of their topics were also found in our questionnaire. In case of future crises, it would be beneficial to conduct further studies on the development and validation of appropriate measurements for young parents and, accordingly, to use these validated instruments in future studies.

## Conclusions

Due to their clinically concerningly high parenting stress levels and particularly high pandemic burden, families with children with crying, sleeping, and feeding problems were a “double risk group” during COVID-19. This might also be true for potential future crises, during which these families, already under immense strain due to their child’s symptomatology, may face compounded challenges. Therefore, it is crucial to address these families in prevention and intervention efforts targeting the reduction of stress. Low-threshold interventions, such as app-based support [42], which can provide accessible and immediate resources, might be promising approaches in this regard. By reducing barriers to professional help, such interventions can offer timely relief and guidance, particularly in the context of strained healthcare systems during crisis situations such as COVID-19. In future crises, decisions regarding restrictions should carefully consider that families with child crying, sleeping, and feeding problems feel very burdened by restricted leisure activities, a lack of support services and social contacts. Therefore, it is essential to prioritize measures that mitigate these pressures. Efforts should include ensuring access to adequate professional support for affected families, even when social distancing measures are implemented. An example of this would be specialized outpatient clinics or trained pediatricians who can offer guidance and support for families struggling with these symptoms. These services can be provided virtually or through safe, in-person consultations, ensuring that families continue to receive the help they need during difficult times. Furthermore, greater emphasis should be placed on the development of safe social connection and leisure activity options, such as virtual playgroups [80]. Additionally, since parenting stress continues to be a concern even after the pandemic [81], it is important to address factors associated with it, such as the increased family conflicts observed during the pandemic. These issues can be effectively addressed through professional counseling, such as by providing conflict management training. Finally, the burden due to increased childcare responsibilities, which was linked to parenting stress in our study, should be addressed by offering support for young families, such as through more flexible working models or the expansion of external childcare options. Large-scale studies are needed to evaluate the long-term effects of such measures on affected families.

## Electronic supplementary material

Below is the link to the electronic supplementary material.

Supplementary Material 1

## Data Availability

The data that support the findings of this study are available from the Chair of Social Pediatrics, Technical University of Munich, but restrictions apply to their availability. Due to the retrospective nature of this study, we were unable to obtain prior consent from patients for the publication of personal raw data. However, anonymized data are available from the authors upon reasonable request. Additionally, the analysis code can be made available upon request to the first author, Michaela Augustin (Michaela.augustin@tum.de).

## Abbreviations

EBI: Eltern-Belastungs-Inventar (German Version of ‘Parenting Stress Index (PSI)’)
COVID-19: Corona Virus Disease 2019
PSI: Parenting Stress Index
