# Supplementary material for: Parenting stress and pandemic burden in families with crying, sleeping, and feeding problems during COVID-19: a case-control study
Source: BMC Psychol. 2025 Apr 17;13:398. doi: 10.1186/s40359-025-02714-z (PMC12007271; doi:10.1186/s40359-025-02714-z)
Supplement: Supplementary file 1 — Supplementary Material 1 [file 40359_2025_2714_MOESM1_ESM.docx]

**Supplement**

| **Table S1**  *Pandemic burden / constraints items* | | |
| --- | --- | --- |
| Variable | Item | Answer |
| Reduced family support services | “How restricted do you currently feel in terms of family support services (e.g. cancellation of baby and children's groups, support services, counseling services, and consultation hours; avoiding courses due to fear of infection)?” | 1 = 'not restricted at all' to 5 = 'very restricted |
| Restricted social contacts of the parents | "How restricted are your private social contacts currently (e.g., with family, friends, acquaintances)? | 1 = 'not restricted at all' to 5 = 'very restricted' |
| Restricted social contacts of the child | "How restricted are your child's social contacts currently (e.g., with caregivers, friends, educational professionals)?" | 1 = 'not restricted at all' to 5 = 'very restricted' |
| Restricted leisure activities | "How restricted are your leisure activities currently (e.g., leisure and sports opportunities, club life, cultural offerings, vacations and travel)?" | 1 = 'not restricted at all' to 5 = 'very restricted' |
| Increased childcare responsibilities | "Has the (planned) childcare situation for your child changed due to the COVID-19 pandemic, so that you currently have more caregiving efforts (e.g., due to daycare closures, loss of care by grandparents, caregiving at home)? | Quantitative:  5-point Likert scale from 1 = not at all to 5 = very much |
| Worries about COVID-19 infections | “Are you currently concerned that you, your child, or someone close to you could get infected?“ | 5-point Likert scale from 1 = not at all to 5 = very much |
| Financial burden due to COVID-19 | “Is there currently a financial burden due to the COVID-19 pandemic?” | 4-point Likert scale from 1 = none to 4 = major |
| Increased family conflicts | “Are there currently more disputes and conflicts in the family?” | 5-point Likert scale from 1 = not at all to 5 = very much |
| Overall pandemic burden | “Taken together, what do you think: how stressful is/was the COVID-19 pandemic for you (please think of measures like social restrictions but also your personal experiences, related worries etc.)?” | 5-point Likert scale from 1 = not at all stressful to 5 = very stressful. |

| **Table S2**  *Pandemic Burden / Constraints – Bivariate Pearson Correlations Between Items Disaggregated by Clinical vs. Nonclinical Group.* | | | | | | | | | |
| --- | --- | --- | --- | --- | --- | --- | --- | --- | --- |
| Variables | 1. | 2. | 3. | 4. | 5. | 6. | 7. | 8. | 9. |
| 1. Reduced family support services |  | .33** | .42** | .28* | .01 | .08 | -.02 | .15 | .29* |
| 1. Restricted social contacts of the parents | .70** |  | .68** | .49** | .09 | .42** | -.03 | .10 | .25* |
| 1. Restricted social contacts of the child | .62** | .71** |  | .45** | .12 | .40** | -.05 | .13 | .28* |
| 1. Restricted leisure activities | .58** | .66** | .53** |  | .30* | .37** | -.36** | .06 | .15 |
| 1. Increased childcare responsibilities | .33** | .39** | .45** | .17 |  | .17 | -.09 | .26* | .06 |
| 1. Worries about COVID-19 infections | .52** | .40** | .38** | .29* | .12 |  | .03 | .25* | .27* |
| 1. Financial burden due to COVID-19 | .33** | .26* | .21 | .16 | .38** | -.07 |  | -.08 | .21 |
| 1. Increased family conflicts | .40** | .28* | .27* | .33** | .19 | .12 | .44** |  | .47** |
| 1. Overall pandemic burden | .50** | .48** | .37** | .36** | .24 | .28* | .25* | .31* |  |

*Note.* The results for the clinical sample are shown below the diagonal. The results for the nonclinical group are shown above the diagonal. **p* < .05 ** *p* < .01
